# A cone-beam computed tomography study of canalis sinuosus and its accessory canals in a South African population

**DOI:** 10.1007/s11282-024-00738-6

**Published:** 2024-02-09

**Authors:** Michael A. Beckenstrater, Mohamed Y. Gamieldien, Chane Smit, Glynn D. Buchanan

**Affiliations:** 1 Department of Odontology, School of Dentistry, Faculty of Health Sciences, University of Pretoria, Pretoria, South Africa; 2 Department of Maxillofacial and Oral Surgery, School of Dentistry, Faculty of Health Sciences, University of Pretoria Oral Health Centre, 31 Bophelo Road, Prinshof Campus, Riviera, Pretoria, 0002 South Africa; 3 Department of Oral and Maxillofacial Pathology, School of Dentistry, Faculty of Health Sciences, University of Pretoria, Pretoria, South Africa

**Keywords:** Canalis sinuosus, Anterior superior alveolar nerve, Accessory canal, Cone-beam computed tomography

## Abstract

**Objectives:**

Canalis sinuosus (CS) is a clinically relevant structure in the anterior maxilla. The present study aimed to determine the prevalence and distribution of CS and its accessory canals (ACs) in the South African population and describe its anatomical variations.

**Methods:**

In total, 500 cone-beam computed tomography (CBCT) scans of the anterior maxilla were assessed for prevalence, sidedness, diameter, and distribution of CS. The frequency, number, diameter, configuration, and point of termination of ACs were also recorded. Statistical analysis was performed using analysis of variance, Kruskal–Wallis, chi-squared, and Fisher Exact tests with P < 0.05.

**Results:**

CS was present in most cases (99.6%), and commonly occurred bilaterally (98.8%). The mean diameter of CS was 1.08 mm (range: 0.50 mm–2.39 mm). Sex, population group, and age had no significant effect on the prevalence or sidedness of CS. Additionally, 535 ACs were observed in 58.8% of the sample, with 42.9% of ACs found bilaterally and 57.1% unilaterally. The mean diameter of the ACs was 0.86 mm on the left and 0.87 mm on the right (range; 0.50 mm–1.52 mm). The majority of ACs maintained a straight vertical configuration (72.3%). ACs most commonly terminated in the anterior palatal region of the maxilla (57.2%). No significant differences were found in any groups mentioned (*P* > 0.05).

**Conclusions:**

A high prevalence of CS as well as ACs were observed in the sample population. Due to their clinical significance, surgical planning with the aid of high quality CBCT scans of the anterior maxilla is advisable.

## Introduction

The anterior maxilla is a site of frequent surgical intervention including the placement of dental implants, orthognathic surgery, endodontic surgery, and the removal of impacted teeth [1–8]. Intimate knowledge of the anatomical variations found in this region is therefore essential. Canalis sinuosus (CS) has been reported as a lesser-known anatomical structure of the anterior maxilla [2, 5]*.* The anterior superior alveolar nerve and artery, housed inside CS, supply the maxillary central incisors, lateral incisors, canines, and surrounding soft tissue with innervation and vascularity [9, 10]. Whilst considered a normal anatomical structure [3, 5, 6, 8, 11, 12], description of CS, along with its variations, is limited in anatomical texts and scholarly literature [3, 5, 7, 11–14].

**Fig. 1:**
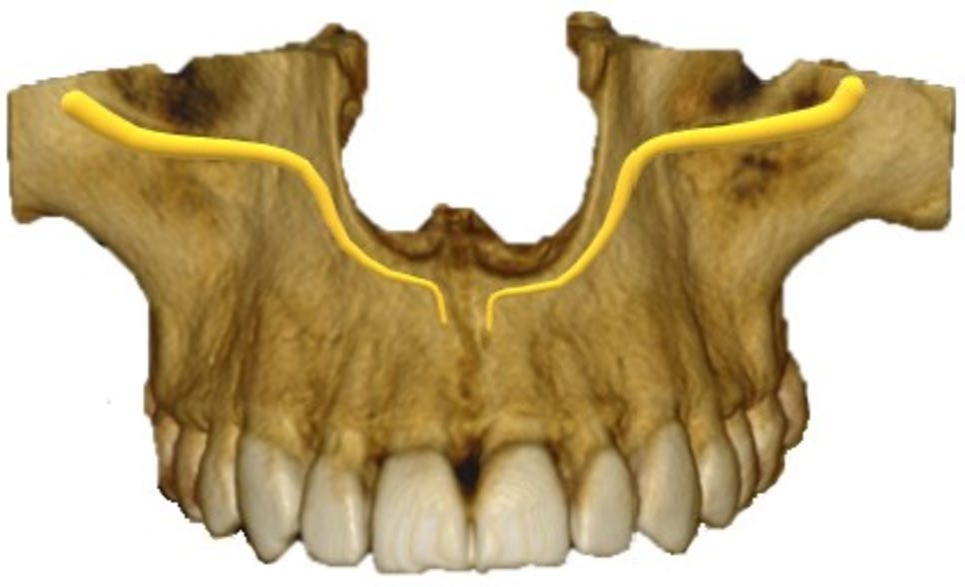
Original 3D rendering of the typical course of a bilateral CS

CS originates from the infraorbital canal posterior to the infraorbital foramen, progressing antero-laterally approaching the anterior wall of the maxilla [1, 2, 10, 14–16]. The canal then changes course, crossing the anterior antral wall, to run medially toward the nasal aperture [1, 2, 10, 14–16]. The canal then descends parallel to the lateral wall of the nasal aperture before terminating adjacent to the nasal septum, anterior to the incisive canal [1, 2, 10, 14–16]. The terminal portion of CS frequently gives rise to accessory intra-osseous canals. These accessory canals (AC) terminate in various anatomical locations, most commonly the anterior palate [3–7, 12, 13, 17–20]. The course of CS is depicted in Fig. 1

Pain and paraesthesia of the surrounding tissues [8, 14, 21–24], intra-operative haemorrhage [8, 24], and failure of implant osseointegration [8, 25], have been reported as consequences of damage to the CS during surgery to the anterior maxilla. Furthermore, CS has been demonstrated as a source of diagnostic uncertainty, mimicking periapical pathology, potentially resulting in endodontic mismanagement [26, 27].

To date, there appears to be no studies detailing the structure and prevalence of CS and its ACs in the South African population. The present study aimed to describe the prevalence and course of CS and its ACs in a South African population by means of retrospective analysis of cone-beam computed tomography (CBCT) scans.

## Materials and methods

### Subjects

This cross-sectional retrospective study evaluated 500 existing CBCT scans in the database of the Pretoria Oral and Dental Hospital, University of Pretoria, South Africa, between July 2017 and July 2021. Scans were assessed chronologically until the required sample size was reached. No new scans were acquired for the purpose of this study.

### Image acquisition

CBCT scans were acquired using a Planmeca Pro-max 3D Max unit (Planmeca Oy, Helsingfors, Finland) and analysed using software of the same manufacturer (Romexis, Planmeca Oy, Helsingfors, Finland). The resolution of the CBCT unit varied between 100 and 600 μm, with 300 to 750 basic frames. This corresponded to the range of voxel sizes (100-600 μm) produced by the unit.

The CBCT unit’s anode current and voltage was set between 8–14 mA and 54–90 kV respectively. The fields of view encompassed the entire maxilla, and the focal spot was set to 0.6 × 0.6 mm in diameter. The exposure settings for each patient were individualised based on the requested field of view and clinical indication. Scans were viewed in a dimly lit room using a 22-inch medical grade monitor (Barco MDRC-222, Kortrijk, Belgium) with a 2-megapixel (1920 × 1080 pixels) resolution.

### Inclusion and exclusion criteria

#### Inclusion criteria:

1. CBCT scans which contained the entire maxilla anterior to the maxillary second premolars bilaterally were included.
2. A maximum voxel size of 400 μm.

#### Exclusion criteria:

1. Partial/unilateral imaging of anterior maxilla.
2. Low technical quality of CBCT scans.
3. Pathological lesions which may have altered the course of CS and its ACs.
4. Missing teeth or implants in the anterior maxilla.
5. Supernumerary or impacted teeth in the anterior maxilla.
6. Clearly visible history of trauma to the anterior maxilla.
7. Clearly visible history of previous surgical intervention in the anterior maxilla.
8. Radiographic artefacts obscuring visualisation of the anterior maxilla.
9. Scans of population groups not classified as “Black African (South African)”, “White” or “Black African (non-South African)” on the hospital file.

### Scan analysis and measurements

All CBCT images were oriented in a standardised and reproducible position to facilitate consistency of analysis and measurement. CBCT scans were examined through means of visual and metric analysis by an investigator, who was calibrated with two experienced dentists. The following parameters were recorded: prevalence, sidedness, diameter, and distribution of CS across demographic data. Furthermore, the prevalence, sidedness, number, mean diameter, configuration, and point of termination of the ACs was also determined.

All CBCT images were examined in axial, coronal, and sagittal planes for the presence of CS according to its typical anatomical description in the literature (depicted in Fig. 1) [1, 2, 9, 15, 17, 18]. Independent bilateral evaluation of the maxilla was performed to determine the prevalence and potential difference in the sidedness of CS. A minimum detection diameter of 0.5 mm was set for the identification of CS and ACs. The diameter of CS was measured along the portion of the canal which ran vertically adjacent to the nasal aperture at its largest diameter.

The terminal portion of CS served as the initial location for detection of ACs in axial, coronal, and sagittal planes. The criteria for identifying an AC included a clearly visible canal which presented as either: (1) a direct extension of CS beyond its typical point of termination, or (2) a direct branch of CS in the case of multiple ACs.

**Fig. 2:**
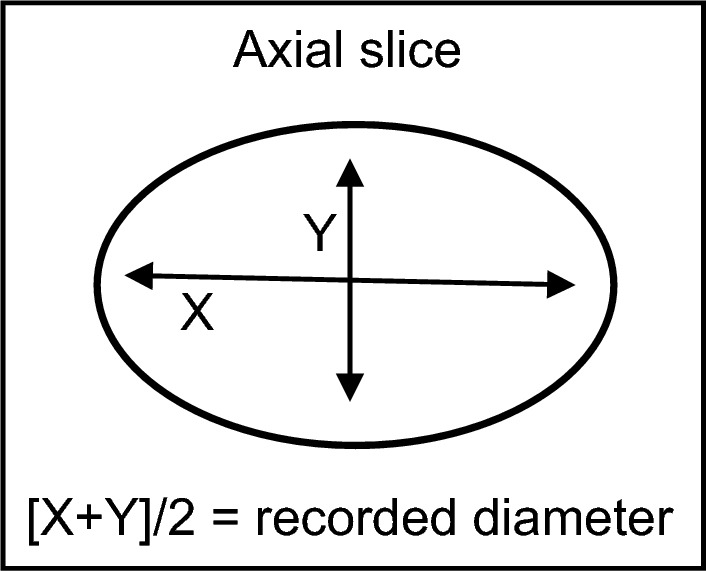
Illustration of the manner in which the diameter of the ACs were measured and recorded

The AC’s diameter was measured in two directions, perpendicular to one another, on the same axial slice. One measurement was taken at the widest point of the AC, and the second measurement at the narrowest point. The average of these two measurements was used as the final recorded diameter for the AC. This was used to compensate for the oval nature of ACs, in keeping with the methodology of Yeap et al*.* [11], demonstrated in Fig. 2.

Termination of the AC was recorded at the point which the AC exited the surface of the maxilla through a foramen. These locations were recorded according to the proximity of the AC foramen relative to local structures (e.g., central incisors, incisive foramen, etc.) as performed in previous studies [3, 5–7, 13, 19]. A qualitative description of the AC’s course was performed based on previous descriptions in the literature [3, 4, 7]. The course of the ACs were classified into the following patterns:

1. Straight vertical – ACs which progressed anteriorly with no medial or lateral deviation in course,
2. Curved medially – ACs with a significant medial deviation in course,
3. Curved laterally – ACs with a significant lateral deviation in course,
4. Curved distally – ACs which turned to progress distally, and
5. Other – ACs which did not fit the other patterns.

### Statistical analysis

Data was captured on a spreadsheet (Microsoft Excel, Microsoft Corporation, Redmond, WA, USA) and statistical analysis performed using SAS Release 9.4 (SAS Institute Inc, Cary, NC, USA). The statistical analysis included both descriptive and inferential statistics. Percentages were compared using chi-squared or Fisher Exact tests depending on the sample size. Values were compared by analysis of variance (ANOVA) and the Kruskal–Wallis test. All statistical tests were two-sided and p values ≤ 0.05 were considered significant.

## Results

### Canalis sinuosus

Most of the subjects in the present study were male (*n* = 308/500, 61.6%), while the remainder were female (*n* = 192/500, 38.4%). Black African (South African) subjects represented 74.6% (*n* = 373/500) of the sample, while White subjects represented 17.0% (*n* = 85/500), and a further 8.4% (*n* = 42/500) of subjects were classified as Black African (non-South African). Subject age ranged between 10 and 89 years, with a mean age of 36.4 years (± 13.9 years).

CS was present in most subjects (*n* = 498/500, 99.6%). In subjects where CS was present, the structure was most frequently observed bilaterally (*n* = 492/498, 98.8%).

The diameter of CS ranged between 0.50 mm-2.39 mm of the left, and 0.50 mm-2.37 mm on the right. While the mean diameter of the CS was recorded at 1.08 mm.

### Effect of demographics on CS

#### Sex:

All females in the study sample presented with CS (*n* = 192/192, 100%), while most males (*n* = 306/308, 99.4%) also presented with CS. Where present, the majority of both males (*n* = 304/306, 99.4%) and females (*n* = 188/192, 97.9%) presented with CS bilaterally.

#### Population group:

All White subjects presented with CS (*n* = 85/85, 100%). Most Black African subjects presented with CS (*n* = 371/373, 99.5%). Where present, the majority of Black African subjects (*n* = 368/371, 99.2%) and White subjects (*n* = 83/85, 97.6%) presented with CS bilaterally.

#### Age:

No significant relationship was found between the presence of CS and age (*P* ≤ 0.05).

An incidental relationship was found between age and the sidedness of CS. However, when compared against a Bonferroni adjusted *P* level of 0.008 the difference was not considered statistically significant, but only of clinical interest.

The effect of age on the presence and sidedness of CS is demonstrated in Tables 1 and 2.

**Table 1.** Prevalence of CS by age

Assessment	Age, years				*P* value*
	** ≤ 20 (%)**	**21–40 (%)**	**41–60 (%)**	** ≥ 61 (%)**	
CS absent	–	2 (0.7)	–	–	1.000
CS present	49 (100)	283 (99.3)	136 (100)	30 (100)	
Total	49 (100)	285 (100)	136 (100)	30 (100)	
Prevalence	**100%**	**99.3%**	**100%**	**100%**	
**95% CI**	**92.7–100%**	**97.5–99.8%**	**97.2–100%**	**88.6–100%**	

*Fisher Exact test

**Table 2.** Sidedness of CS by age

Sidedness	Age, years				*P* value*
	** ≤ 20 (%)**	**21–40 (%)**	**41–60 (%)**	** ≥ 61 (%)**	
Unilateral **	1 (2.0)	3 (1.1)	–	2 (6.7)	0.029
Bilateral	48 (98.0)	280 (98.9)	136 (100)	28 (93.3)	
Total	**49 (100)**	**283 (100)**	**136 (100)**	**30 (100)**	

*Fisher Exact test

** Patients ≤ 20 years of age: Left 0 (0.0%), Right 1 (2.0%)

Patients 21–40 years of age: Left 1 (0.4%), Right 2 (0.7%).

Patients 41–60 years of age: Left 0 (0.0%), Right 0 (0.0%).

Patients ≥ 61 years of age: Left 0 (0.0%), Right 2 (6.7%)

### Accessory canals

A total of 535 ACs were observed, 49.9% (*n* = 267/535) were found on the left and 50.1% (*n* = 268/535) on the right. The highest number of ACs observed in a single patient was seven. ACs were found in 58.8% (*n* = 294/500, 95% CI: 54.4–63.0%) of cases, and not seen in the remaining 41.2% (*n* = 206/500). In cases where ACs were found 42.9% (*n* = 126/294) occurred bilaterally and 57.1% (*n* = 168/294) occurred unilaterally. Similar numbers of ACs occurred unilaterally on the left (*n* = 85/294, 28.9%) or right (*n* = 83/294, 28.2%).

When present, the diameter of the ACs ranged between 0.50 mm and 1.28 mm on the left, and 0.50 mm–1.52 mm on the right. The mean diameter of the ACs were recorded at 0.86 mm (± 0.28) on the left and 0.87 mm (± 0.27) on the right.

**Fig. 3:**
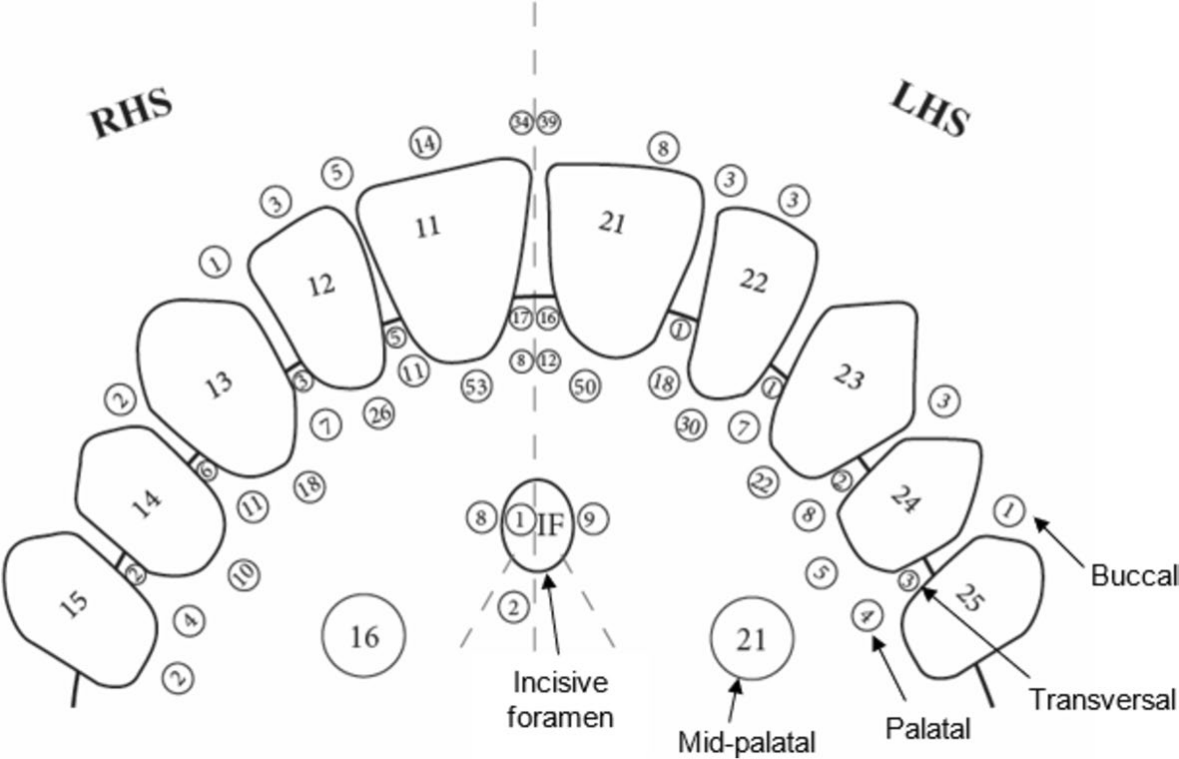
Illustration of the points of termination of the ACs found in the present study divided into five principal regions, namely; buccal, transversal, palatal, near the incisive foramen, and mid-palatal. Diagram based on the work of Machado et al. [6]. RHS—right hand side, LHS—left hand side, numbers within teeth correspond with the FDI tooth numbering system, numbers within circles represent the number of ACs found terminating at each point

The majority of ACs terminated in the anterior palatal region accounting for 57.2% (*n* = 306/535) of all detected ACs. A substantial portion (*n* = 116/535, 21.7%) of the ACs occupied a buccal position, with 10.5% (*n* = 56/535) found in a transversal position. A small number of ACs terminated in the mid-palatal region (*n* = 37/535, 6.9%) with even fewer terminating near the incisive foramen (*n* = 20/535, 3.7%). Greater detail surrounding the point of termination of the ACs can be seen in Fig. 3.

**Fig. 4:**
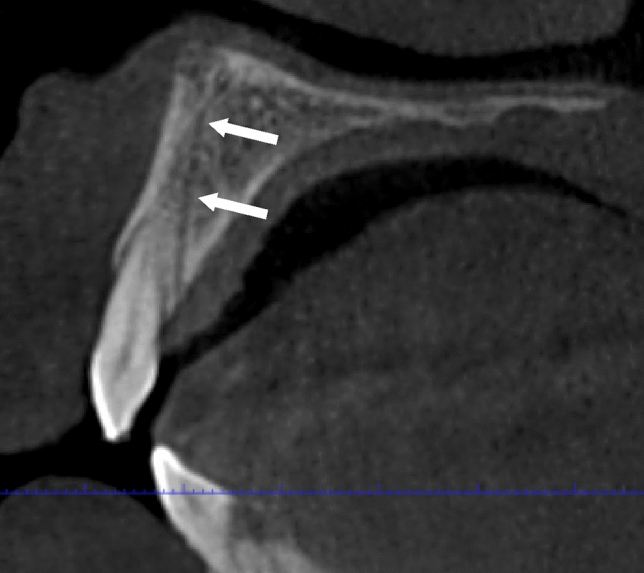
Demonstrates an example of an AC of CS occupying a straight vertical position indicated by the white arrows

**Fig. 5:**
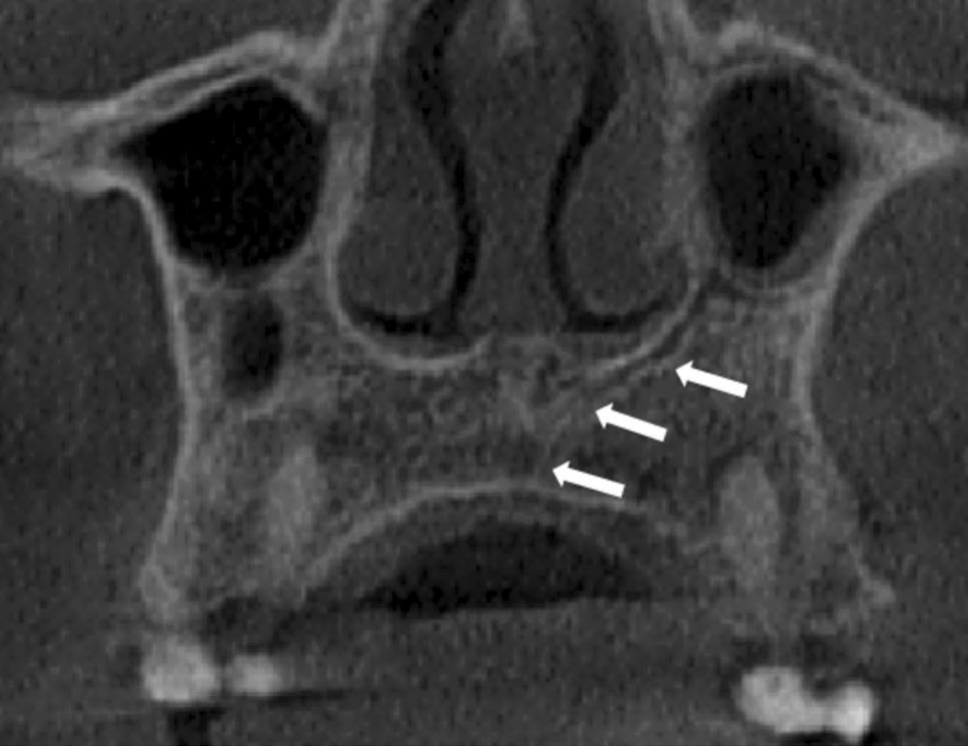
Demonstrates an example of an AC of CS occupying a curved medial configuration indicated by the white arrows

**Fig. 6:**
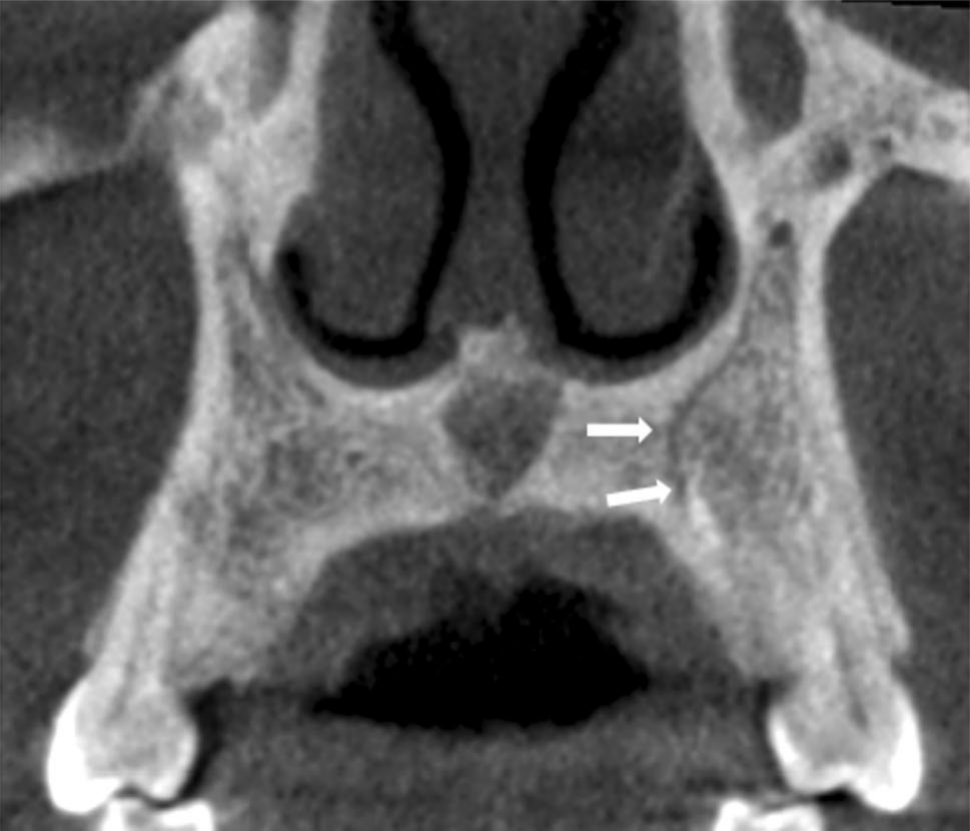
Demonstrates an example of an AC of CS occupying a curved lateral configuration indicated by the white arrows

**Fig. 7:**
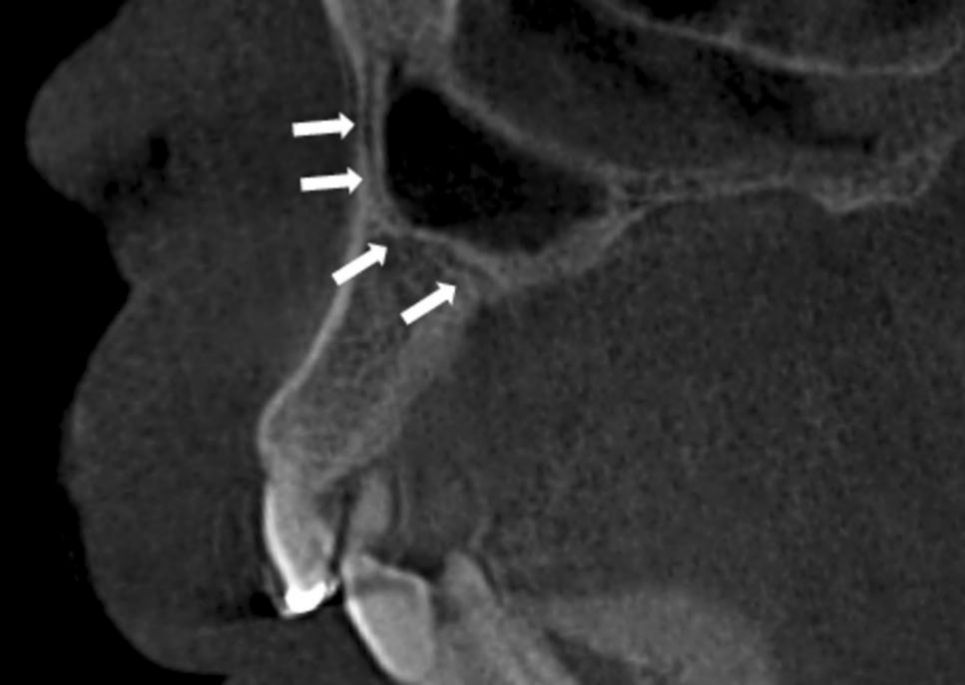
Demonstrates an example of an AC of CS occupying a curved distal configuration indicated by the white arrows

**Fig. 8:**
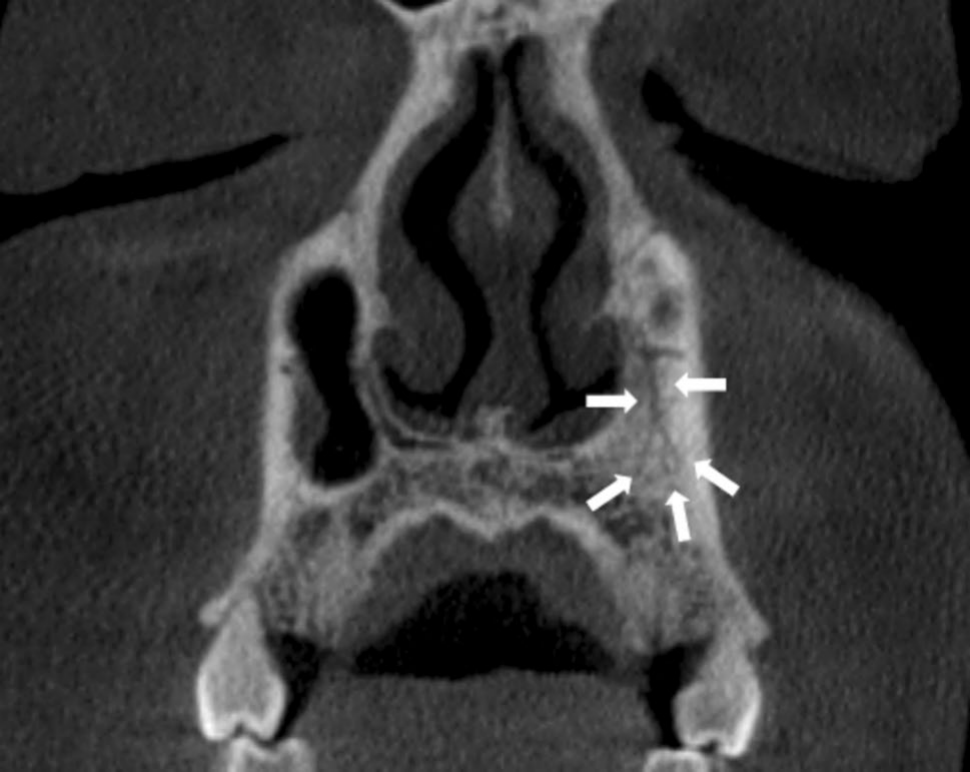
Example of an AC classified as an anatomical anomaly. The AC on the LHS of the maxilla branches off the horizontal aspect of CS and descends vertically before splitting into multiple branches inferiorly indicated by the white arrows

The most common AC configuration was the straight vertical which represented 72.3% (*n* = 387) of ACs, followed by curved distally 12.7% (*n* = 68). ACs which curved laterally and curved medially represented 7.5% (*n* = 40) and 7.3% (*n* = 39) respectively. One case did not fit the given descriptions and was reported as “other” (*n* = 1/535, 0.2%). No significant differences were found in the course of the ACs between the left and right sides. These findings are reported in greater detail in Table 3. Figures 4, 5, 6, 7 and 8 demonstrate various AC configurations.

**Table 3.** Course of the AC

Classification	Left (%)	Right (%)	*P* value*
Straight vertical (387; 72.3%)	194 (72.6)	193 (72.0)	0.923
Curved medially (39; 7.3%)	20 (7.5)	19 (7.1)	0.870
Curved laterally (40; 7.5%)	19 (7.1)	21 (7.8)	0.869
Curved distally (68; 12.7%)	33 (12.4)	35 (13.1)	0.897
Other (1; 0.2%)	1 (0.4)	–	0.499
Total **(535; 100%)**	**267 (100)**	**268 (100)**	

* Fisher Exact test

## Discussion

Modern dental treatment has seen a rise in the use of CBCT due to improved scan quality, cost efficiency, and reduced radiation exposure [28]. Ferlin et al*.’s* review of literature suggested CBCT scans to be the best modality for the evaluation of CS, where conventional radiographic techniques (periapical and panoramic radiographs) proved less effective [8]. Many studies investigating CS and its ACs have evaluated CBCT scans [1, 2, 5–7, 11–13, 17–19, 25, 29, 30].

The CS exists as a distinct anatomical entity with reported prevalence ranging from 66.5% to 100% in different population groups [1, 2, 8, 11, 12, 18, 19, 30]. The bilateral presence of CS has been reported to range between 46 and 100% [1, 2, 11, 12, 18, 19]. The present study found that the vast majority of South African patients (99.6%) presented with CS. When present, it frequently occupied a bilateral distribution (98.8%). Variation in the prevalence and sidedness of CS may be attributed to inherent differences between population groups, or differing study designs and methodology. When reporting the prevalence of CS, Wanzeler et al*.*, Ghandourah et al*.*, and Gurler et al*.* differentiated between CS and its ACs, thus observing a higher prevalence of the structure (88%, 100%, 100%, and 99.6% respectively) [1, 2, 18]. Other authors, such as Aoki et al*.* and Anatoly et al*.* regarded CS and its ACs as a single structure and reported CS as absent if no ACs were present. This differing methodology may have contributed to a lower recorded prevalence of CS in these studies (66.5% and 67% respectively) [12, 19].

The present study found the mean diameter of CS to be 1.08 mm on both the right and left sides. This measurement is comparable to the mean diameters of CS reported by Gurler et al*.* and Sedov et al*.* in Turkish and Russian populations at 1.37 mm and 0.95 mm respectively [2, 31]. The range of diameters recorded by Gurler et al*.* (0.75 mm-2.25 mm) and Sedov et al*.* (0.3 mm-2.1 mm) is similarly comparable to that of the present study (05 mm-2.39 mm) [2, 31]. Differences in the reported diameters between studies may be attributed to the varying minimum detection diameter threshold selected (Gurler et al*.*: 0.75 mm [2], Sedov et al*.*: none [31], and the present study: 0.5 mm). de Oliveria-Neto et al*.*, suggested that comparison of the mean diameters of CS in different populations is difficult due to a lack of available data and differences in study methodologies [30].

No relationships regarding the prevalence or sidedness of CS and sex were found in the present study. This is consistent with studies conducted in Brazilian, Belgian, Turkish, German, Chinese, Australian, and Cypriot populations [1, 3, 5, 7, 11, 17, 18, 29]. Age was also shown to have no effect on the presence or sidedness of CS, in keeping with studies of Brazilian, Turkish, Russian, and Chinese populations [1, 6, 7, 12, 13, 17, 19]. Furthermore, the present study found that ethnicity in the South African context had no influence on the presence or sidedness of CS. These findings suggests that CS should routinely be observed bilaterally—irrespective of age, sex, or population group.

The prevalence of ACs varies substantially between different populations, with ranges reported between 8.17 and 100% [5–7, 11–13, 17–19, 29] (Table 4). More than half the South African population (58.8%) presented with at least one AC, in keeping with the findings of Machado et al*.* in a Brazilian population [6]. This finding however differed notably from other populations [5, 7, 11–13, 17–19, 29]. Generalisation regarding the prevalence of the ACs can therefore not be made. The differences in the prevalence of ACs may represent true anatomical differences between populations based on genetic, ethnic, and geographical factors [3, 32]. Alternatively, such differences may be attributed to inconsistencies in study design, including; minimum detection diameter threshold, inclusion criteria, CBCT machine, software, voxel size, slice thickness, and the investigators experience [29]. Şalli and Öztürkmen recorded the lowest prevalence of ACs (8.17%) but used a larger minimum detection diameter [13] than other studies [6, 11, 18, 29]. Beyzade et al. recorded the highest prevalence of ACs (100%) in a Cypriot population, however, evaluated the smallest sample (*n *= 91) which may have influence the results [29].

**Table 4.** Summary of the prevalence of ACs across different populations ranked in descending order

Study	Population	Sample size (n)	AC Prevalence (%)
Beyzade et al*.* [29]	Cypriot	91	100.0
Orhan et al*.* [5]	Turkish	1460	70.8
Ghandourah et al*.* [18]	German	201	67.6
The present study	South African	**500**	**58.8**
Machado et al. [6]	Brazilian	1000	52.1
Shan et al*.* [17]	Chinese	1007	36.9
Tomrukçu & Köse [7]	Turkish	326	34.7
Şalli & Öztürkmen [13]	Turkish	673	8.2

The ACs of the present study recorded a mean diameter of less than 1.0 mm. This was smaller than the mean diameters found in Brazilian, Turkish, and Chinese populations respectively [6, 7, 17]. These studies reported mean diameters of 1.19 mm, 1.07 mm, and 1.1 mm respectively [6, 7, 17]. Yeap et al*.* reported a substantial difference between the widest (1.08 mm) and narrowest (0.71 mm) diameters of CS in the Australian population [11]. This study furthermore established the oval nature of CS [11]. The mean diameter of the ACs recorded in the present study may be attributed to the use of a smaller minimum detection threshold when compared to others [6, 17]. Yeap et al. however suggested the diameter of ACs to vary within single canals and between subjects [11]. Variation of mean diameter seen between different populations may therefore be due to differences in study design as opposed to true anatomical differences.

Previous investigations found ACs most commonly terminated in the anterior palatal region of the maxilla (91.1% and 76% respectively) [6, 19]. Less common locations include buccal (5.1% and 12%) and transversal (3.8% and 12%) positions [6, 19]. The present study found fewer ACs terminating in a palatal position (57.2%), while more occupied buccal (21.7%) and transversal (10.5%) positions. This may be attributed to the use of a greater number of AC termination locations used in the present study. The most common points of AC termination in the present study were palatal of the central incisors and buccal of the interproximal region between the central incisors. This was consistent with the findings of previous investigations which found that ACs most commonly terminated in this region [5, 11, 12, 18].

The course of the ACs is poorly documented in the literature [7]. In the present study, all ACs progressed through the maxilla from a superior point of origin to an inferior point of termination. The majority of the ACs demonstrated a straight vertical configuration (72%). Yeap *et* al. reported the potential for complex AC configurations, with some cases displaying convergence or various branching patterns [11]. The present study did not find many of these complex configurations.

CS is a clinically significant structure which may affect surgical procedures in implantology, oral surgery, endodontics, and periodontology [8, 21–24, 26, 27]. The most frequently reported clinical complication associated with damage to CS is post-operative pain and paraesthesia [21–23]. The possibility of severe intra-operative haemorrhage and failure of dental implant osseointegration has additionally been reported following damage to CS [8, 21–23]. Furthermore, CS has proven to be a source of diagnostic uncertainty, in some instances resembling periapical pathology with the potential to lead to endodontic mismanagement [26, 27]. The high prevalence and complex anatomy of CS and ACs found in the present study supports the recommendation of routine CBCT scanning and evaluation prior to surgical intervention in the anterior maxilla, in order to reduce the risk of complications [11, 25].

## Conclusion

CS is a distinct anatomical entity which is present bilaterally in most Southern African subjects. The ACs displayed highly variable anatomy, most frequently demonstrating a straight vertical configuration, and terminating in the anterior palatal region of the maxilla. Evaluation of a high quality CBCT scan prior to surgical intervention of the anterior maxilla may be beneficial in the avoidance of intra-operative damage to CS and its ACs. A thorough understanding of clinically relevant anatomy of the anterior maxilla is essential to avoid surgical complications in this region.

## Data Availability

All collected data is available online from the University of Pretoria’s research repository (UPSpace).
